# A neuromusculoskeletal modelling approach to bilateral hip mechanics due to unexpected lateral perturbations during overground walking

**DOI:** 10.1186/s12891-023-06897-7

**Published:** 2023-10-02

**Authors:** Yunchao Zhu, Ji Huang, Xin Ma, Wen-Ming Chen

**Affiliations:** 1 Academy for Engineering and Technology, Fudan University, 220 Handan Rd., Shanghai, 200433 China; 2 National Clinical Research Center for Geriatric Diseases (NCRCGD), Huashan Hospital Affiliated to Fudan University, No.12, Wulumuqi Middle Rd., Shanghai, 200040 China

**Keywords:** Balance, Biomechanical analysis, Musculoskeletal model, Perturbation, Overground walking

## Abstract

**Background:**

Current studies on how external perturbations impact gait dynamics have primarily focused on the changes in the body's center of mass (CoM) during treadmill walking. The biomechanical responses, in particular to the multi-planar hip joint coordination, following perturbations in overground walking conditions are not completely known.

**Methods:**

In this study, a customized gait-perturbing device was designed to impose controlled lateral forces onto the subject’s pelvis during overground walking. The biomechanical responses of bilateral hips were simulated by subject-specific neuromusculoskeletal models (NMS) driven by in-vivo motion data, which were further evaluated by statistical parameter mapping (SPM) and muscle coactivation index (CI) analysis. The validity of the subject-specific NMS was confirmed through comparison with measured surface electromyographic signals.

**Results:**

Following perturbations, the sagittal-plane hip motions were reduced for the leading leg by 18.39° and for the trailing leg by 8.23°, while motions in the frontal and transverse plane were increased, with increased hip abduction for the leading leg by 10.71° and external rotation by 9.06°, respectively. For the hip kinetics, both the bilateral hip joints showed increased abductor moments during midstance (20%-30% gait cycle) and decreased values during terminal stance (38%-48%). Muscle CI in both sagittal and frontal planes was significantly decreased for perturbed walking (*p* < 0.05), except for the leading leg in the sagittal plane.

**Conclusion:**

The distinctive phase-dependent biomechanical response of the hip demonstrated its coordinated control strategy for balance recovery due to gait perturbations. And the changes in muscle CI suggested a potential mechanism for rapid and precise control of foot placement through modulation of joint stiffness properties. These findings obtained during actual overground perturbation conditions could have implications for the improved design of wearable robotic devices for balance assistance.

**Supplementary Information:**

The online version contains supplementary material available at 10.1186/s12891-023-06897-7.

## Background

Falls-related injuries most frequently occur in individuals with balance disorder during the weight-transferring phase in gait [1], which raises significant concern for the healthcare community. And gait stability in the mediolateral direction requires more active control, involving the bilateral feet to establish a sufficient base of support (BoS) to prevent excessive Center of Mass (CoM) excursion during step-to-step transitions [2]. Current approaches for enhancing balance function include body weight-supporting balance training and perturbation-based gait training. The body weight-supporting device employs an overhead suspension system for partial body weight support to facilitate walking in patients with neurological conditions [3]. Perturbation-based gait training focuses on improving gait stability in individuals with balance function degeneration through exposure to perturbations [4]. More recently, exoskeletons have emerged as a promising technique for balance assistance during rehabilitation training. Those are often incorporated with sensors for continuous monitoring of the wearer's gait stability, and utilize powered actuators to modulate one’s foot placement by delivering controlled resistance or assistance at lower extremity joint during the swing phase [5]. However, both robotic assistive studies directly focused on the modulation between the CoM states and foot placement during perturbation, neglecting the crucial involvement of the hip in the execution of stepping in response to perturbation [6]. This neglect may impede human–machine coordination resulting in uncomfortable interaction, highlighting the necessity of exploration into the hip adaptive changes due to perturbation in gait.

Previous studies have revealed the prominent role of the hip joint in adaptive balance control. For instance, healthy subjects exhibit a preference for shorter, faster, and wider steps in response to perturbations [7], induced by the Computer Assisted Rehabilitation Environment (CAREN) system, which includes a split-belt treadmill and a 6-degree-of-freedom robotic motion platform. And this protective stepping primarily relies on hip motion in the frontal plane, which was considered as the effective balance control mechanism to increase the margin of stability (MoS) determined by the distance between CoM states and BoS [8]. In addition, age-related decline in the kinetic response of the hip joint, including the reduction of peak joint moment and rate of such moment generation [9], could significantly undermine the mediolateral balance control during the step-to-step transition phase in gait [10]. In terms of muscle activations, the hip abductor muscle (gluteus medius) was proved to be highly associated with lateral foot placement [11], and there existed a phase delay of gluteus medius activation following lateral perturbation [12]. The abnormal changes in hip muscle activation confirmed the active balance control of the hip in the mediolateral direction [2].

While the above studies have shed some light on balance mechanisms due to lateral perturbations, those are subjected to the following limitations. First, existing perturbation protocols to provoke adaptive responses mostly are based on the treadmill [7, 11]. However, whether treadmill walking induced identical motor response as compared to overground walking remains controversial [13, 14]. Studies have shown that during treadmill walking, individuals tend to adopt a "cautious gait" characterized by shorter step lengths and reduced backward trunk lean [15]. Moreover, significant differences have been observed between overground and treadmill groups for perturbation-based balance training outcomes [16]. Therefore, implementing perturbations during overground walking may represent a more physiological-meaningful approach to understanding hip adaptive responses to gait perturbation. In addition, previous studies have primarily concentrated on a particular time instant in gait, such as the peak hip abductor’s moment or peak joint angles as stated above. However, the response of the bilateral hip joints during gait is considered to be highly phase-dependent. A recent study has suggested that step-to-step adjustments occur predominantly during push-off and early swing phases after perturbation [8]. Moreover, investigating changes in muscle activation could provide further insights into the neuromuscular control mechanism of the hip. But prior studies mostly focused on single superficial muscle activation, and few studies evaluated muscle coactivation patterns induced by external perturbations due to the narrow surface area exposed by muscle bundles [17]. Some studies suggested that increased joint muscles’ CI could be considered as a compensation mechanism to increase joint stiffness for reducing the risk of potential injury [18]. Compared to young healthy adults, the elderly showed higher muscle coactivation in waking [19]. However, higher muscle coactivation could reduce the performance and flexibility of joint movement which constrain the execution of voluntary responses to perturbation [20, 21].

Neuromusculoskeletal model (NMS) has emerged as the pre-eminent technique for evaluating internal joint mechanics and assessing alterations in muscle function across all three anatomic planes [22, 23]. And it has been applied to many fields with great potential, including the evaluation of active orthotics [24], ergonomic optimization [25], and performance assessment of orthopedic implants [26]. Previous studies have indicated a sufficient level of consistency in lifting, inclined walking and running [23, 27]. However, none of the previous studies have verified the feasibility of the NMS during challenging (perturbed) gait conditions.

Therefore, the purpose of the study was to investigate the adaptive mechanism of the hip in balance recovery due to external perturbations during overground walking. The study also aimed to verify the feasibility of utilizing subject-specific neuromusculoskeletal modeling (NMS) for biomechanical analysis during perturbed walking conditions.

## Methods

### Perturbation experiments

**Fig. 1:**
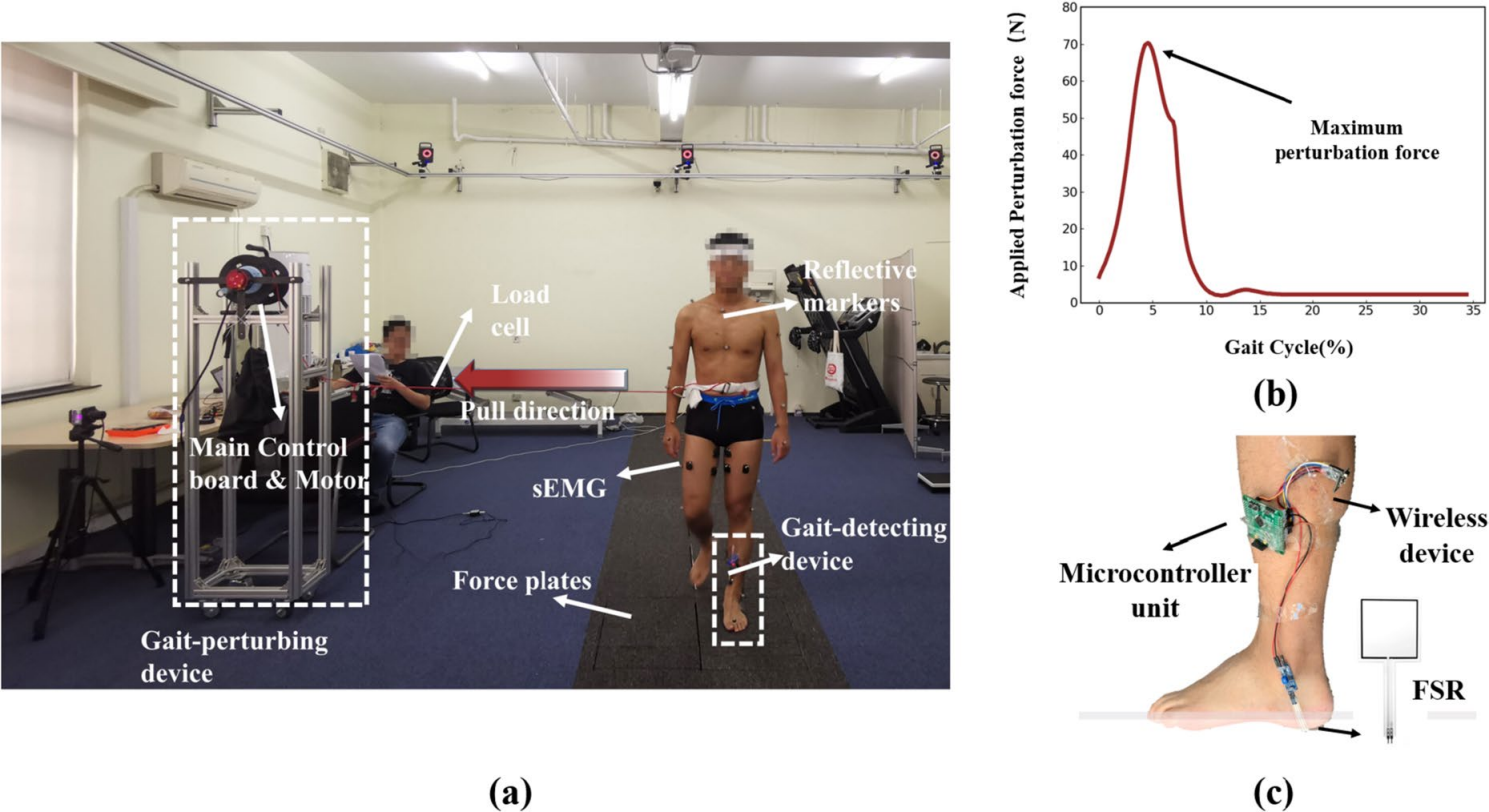
Experiment setup in the motion capturing lab with a customized gait-perturbing device (**a**), the measured perturbation forces (**b**), and the electronic design of the gait event detection unit (**c**)

The perturbation experiment was performed by utilizing a custom-designed gait-perturbing device. A brushless servo motor was fixed onto the mechanical frame, and perturbation force was applied to subjects’ pelvis through transmission mechanism (see Fig. 1a). The magnitude and duration of the perturbation force were controlled by the main control board, and real-time perturbation force data were measured by the tension load cell (see Fig. 1b). A wireless microprocessor controller was devised to detect gait event of the leading leg by monitoring changes in plantar pressure using a force-sensing resistor (FSR) attached at plantar heel region (see Fig. 1c). To reduce mechanical response delay, admittance control was used to keep transmission mechanism at a pre-tension state before imposing perturbation forces, and the duration was limited in loading response phase at 90 ms. Perturbations were applied to the subjects' pelvis to induce controlled alterations in the CoM during walking [28]. The magnitude of the perturbation force was set equal to 8% of the subjects’ body weight and tested before the experiment to ensure safety and feasibility. The perturbed walking group and unperturbed walking group (zero perturbation force) were randomly imposed onto the subject’s pelvis to minimize the subject’s anticipatory postural adjustments. The trial that induced multistep response would not be included in the analysis. The mechanical structure of the gait perturbing device and its corresponding control code are included as supplementary materials.

Five healthy young adults volunteered to participate in this study [Male; age = 23 ± 1yr, height = 1.70 ± 0.09 m, mass = 63.8 ± 12.1 kg (mean ± SD)], and informed consent was obtained. This study was conducted in accordance with the Declaration of Helsinki, approved by the ethics committee of the regional hospital and registered with ChiCTR.org.cn (www.chictr.org.cn, 17/12/2021, ChiCTR2100054453). All trials were performed in a Motion Capture lab utilizing eight cameras (see Fig. 1) and plug-in-gait full body model marker protocol (Vicon Nexus 1.8.5, VICON, Oxford Metrics Ltd, UK) (see Fig. 1). Skin marker data was sampled at 100 Hz and filtered by fourth-order, zero-lag Butterworth filters, with a cut-off frequency of 6 Hz respectively. Ground reaction force sampled at 1080 Hz was simultaneously recorded from four AMTI force plates (Advanced Mechanical Technology Inc, Watertown, MA, USA). Electromyography signals from four muscles(rectus femoris, biceps femoris, gluteus medius and adductor longus) were recorded using a wireless device(Delsys Inc, Boston, USA) and sampled at 1000 Hz. Subsequently, the recorded signals were rectified and filtered using a fourth-order Butterworth filter with a cutoff frequency of 6 Hz. Participants walked at self-selected walking speed (averaged at 1.0 ± 0.15 m/s), taking at least 8 steps. A perturbing device was placed in the middle of the walkway for the study. To mitigate the influence of initial gait on the experiment, subjects were instructed to start their walk at least three steps away from the experimental area. Each subject performed 40 trials, including 20 normal walking trials and 20 perturbed walking trials.

### Model analysis

**Fig. 2:**
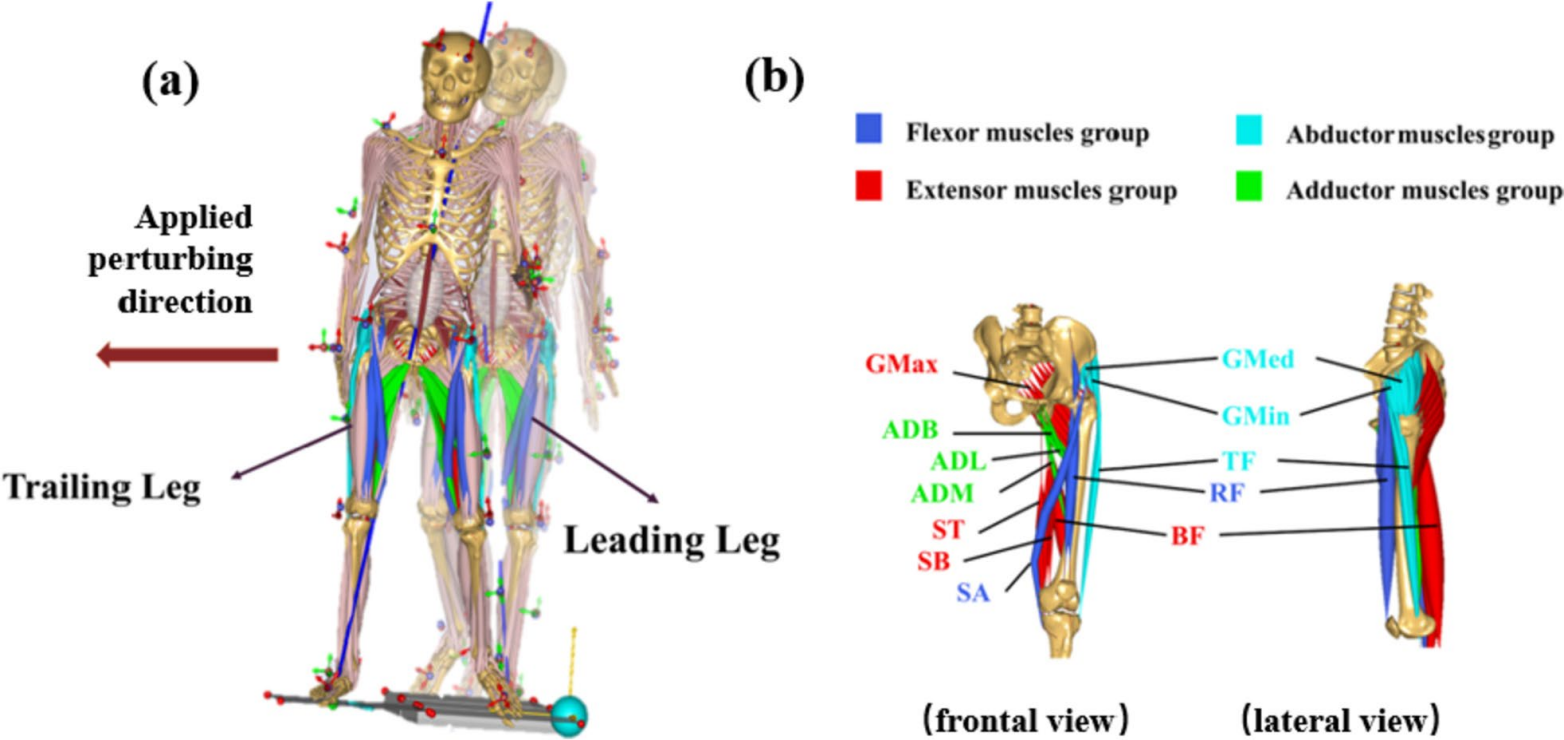
The subject-specific neuromusculoskeletal models (NMS) developed for adaptive responses analysis due to controlled perturbing forces (**a**), and grouped major muscle bundles for controlling hip motions in different anatomical planes (**b**)

**Fig. 3:**
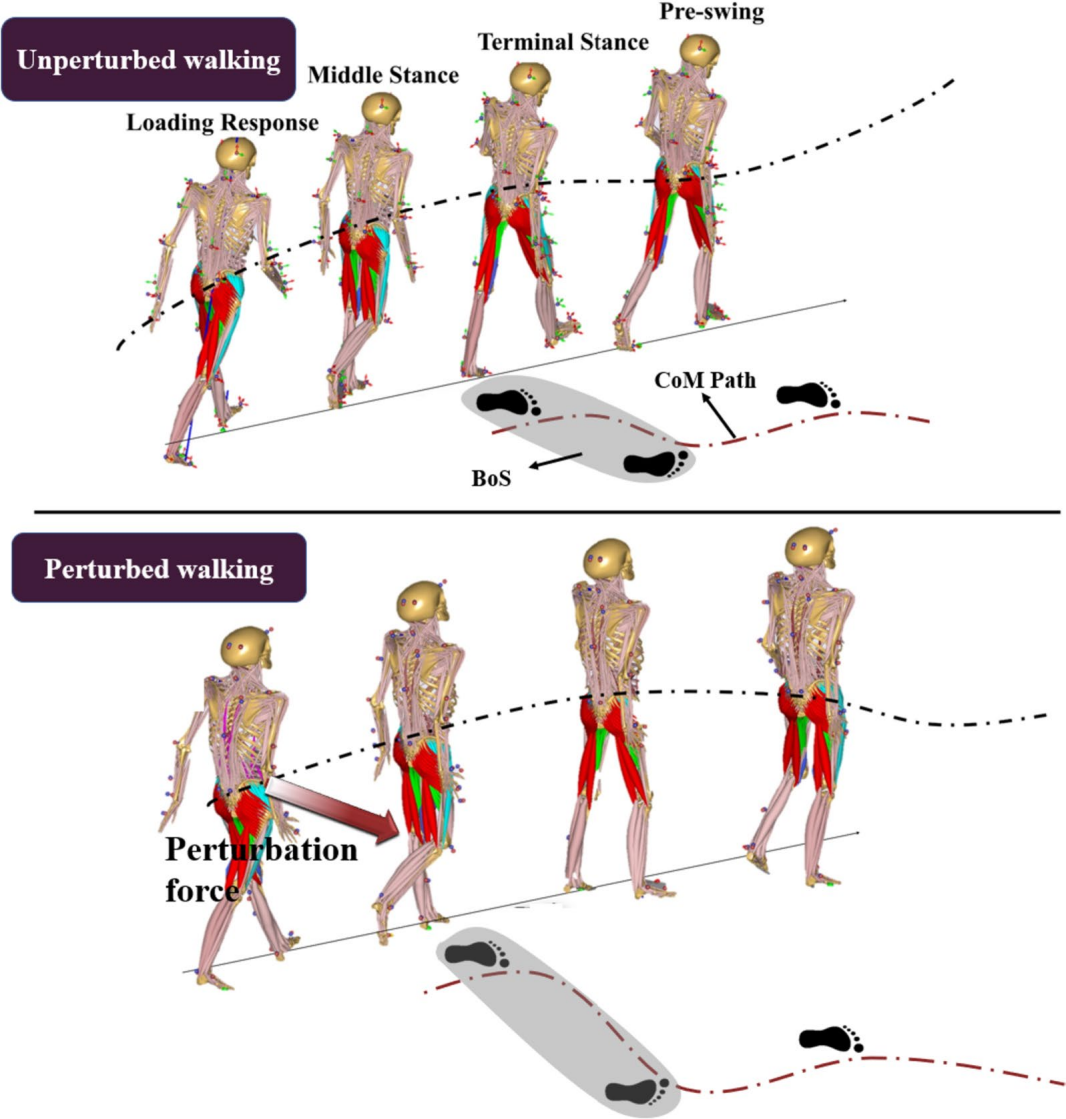
Visualization of neuromusculoskeletal simulations driven by in-vivo motion data during perturbed and unperturbed walking

The neuromusculoskeletal modelling (NMS) offers a powerful computational method to investigate the muscle functions of the body system through inverse dynamic analysis. However, a generic model will not meet the experimental requirement for joint reaction force calculation due to the unappropriated estimation of the subject’s anthropometric data. Thus, the current subject-specific musculoskeletal model was modified based on the GaitFullBody model from Anybody modeling system (AnyBody Technology, Denmark) for model repository with 42 degrees of freedom (see Fig. 2a), which is one of the most dedicated full-body musculoskeletal models. Parameters optimization and segments’ scaling were performed to match the subject’s morphology to reduce a global error metric between experimental and virtual motion marker positions before performing the simulation. Mass-fat scaling algorithm was adapted to estimate each individual’s segment length and mass, according to the measured anthropometric data (body weight, body height, pelvis width, thigh, shanks, and foot length). The subject-specific neuromusculoskeletal model was driven by in-vivo motion data obtained through the motion capture system, enabling the capturing of dynamic motor variations during perturbed and unperturbed walking conditions (see Fig. 3).

In this study, the Hill-type muscle model was employed to characterize all skeletal muscles. Muscle redundancy problem and muscle activations were solved and calculated by inverse dynamics approach and third-order polynomial muscle recruitment algorith [29], which calculated as follows:

1 $$\begin{array}{lll}Min & G\left(f^{\left(M\right)}\right)\\s.t.&\mathbf{Cf}=\mathbf{d}\\&f^{\left(M\right)}_i\geq\mathbf0,i\,\upepsilon\{1,2,...,n^{(M)}\}\end{array}$$

2 $$G(f^{(M)})=\sum_i\left(\frac{f^{(M)}_i}{N_i}\right)^p$$

where $\mathrm{G}$ represents the optimal objective function. ${f}^{(M)}$ is the muscle force, which together with joint reaction force constitutes a n-dimensional vector f. C represents the system’s coefficient matrix associated with anatomy, and the vector d denotes external force. ${N}_{i}$ are the normalizing factors, typically muscle strength.

Joint moments were normalized by the subject’s mass(M), leg length(L), and graviton acceleration(g). For validating the muscle dynamics, a comparison between model-simulated and experimentally-measured muscle electromyographic signals was conducted. Specifically, we compared the normalized muscle activation levels of four major surface muscle bundles, including hip flexor (rectus femoris), extensor (biceps femoris), abductor (gluteus medius) and adductor (adductor longus) in unperturbed walking and perturbed walking. Pearson’s correlation coefficients (r) were used to evaluate the relationships between the two methods.

To better analyze the gait phase-dependent joint responses, T-tests with SPM (statistical parameter mapping) were employed to identify gait phases, where joint responses had significant differences (*p* < 0.05) between the unperturbed and perturbed walking conditions. The SPM method would allow a time-wise continuous analysis for the significant changes in hip kinematics and kinetics for the entire gait cycle. [30]. The hip kinematics and kinetics adhered to the guidelines of the International Society of Biomechanics [31].

Muscle coactivation index (CI) was calculated by identifying the overlap between the agonist and antagonist muscle activation curves [32], which were post-processed following the calculations of the muscle activation levels by the musculoskeletal mode. The calculation method was as follows:

3 $$CI=100\times\frac{\int^{t_2}_{t_1}\text{min}[EMG(t)_\text{M1},\,EMG(t)_\text{M2}]dt}{\int^{t_2}_{t_1}\text{max}[EMG(t)_\text{M1},\,EMG(t)_\text{M2}]dt}$$

where ${M}_{1}$ is agonist muscle group and ${M}_{2}$ is antagonist muscle. In this study, iliacus, rectus femoris (RF) and sartorius (SA) were selected as hip flexor muscles, gluteus maximus (GMax), biceps femoris (BF), semitendinosus (ST), semimembranosus (SB) as hip extensor muscles, gluteus medius (GMed), gluteus minimus (GMin) and tensor fascia (TF) as abductor muscles, adductor magnus (ADM), adductor longus (ADL) and adductor brevis (ADB) as adductor muscles (see Fig. 2b). And ${t}_{1}$ denoted the time of leading leg heel strikes and ${t}_{2}$ denoted the time of the trailing leg consecutive heel strikes in the current study. The time of consecutive heel strikes is the step time when the new BoS is formatted. We utilized paired t-tests for normally distributed data and the Wilcoxon rank sum test for non-normally distributed data to compare results between perturbed and unperturbed walking conditions. And the normality of the data was verified with the Shapiro–Wilk test before performing paired t-tests (see supplementary material). The level of statistical significance was set at *P* < 0.05.

## Results

### Model validation

**Fig. 4:**
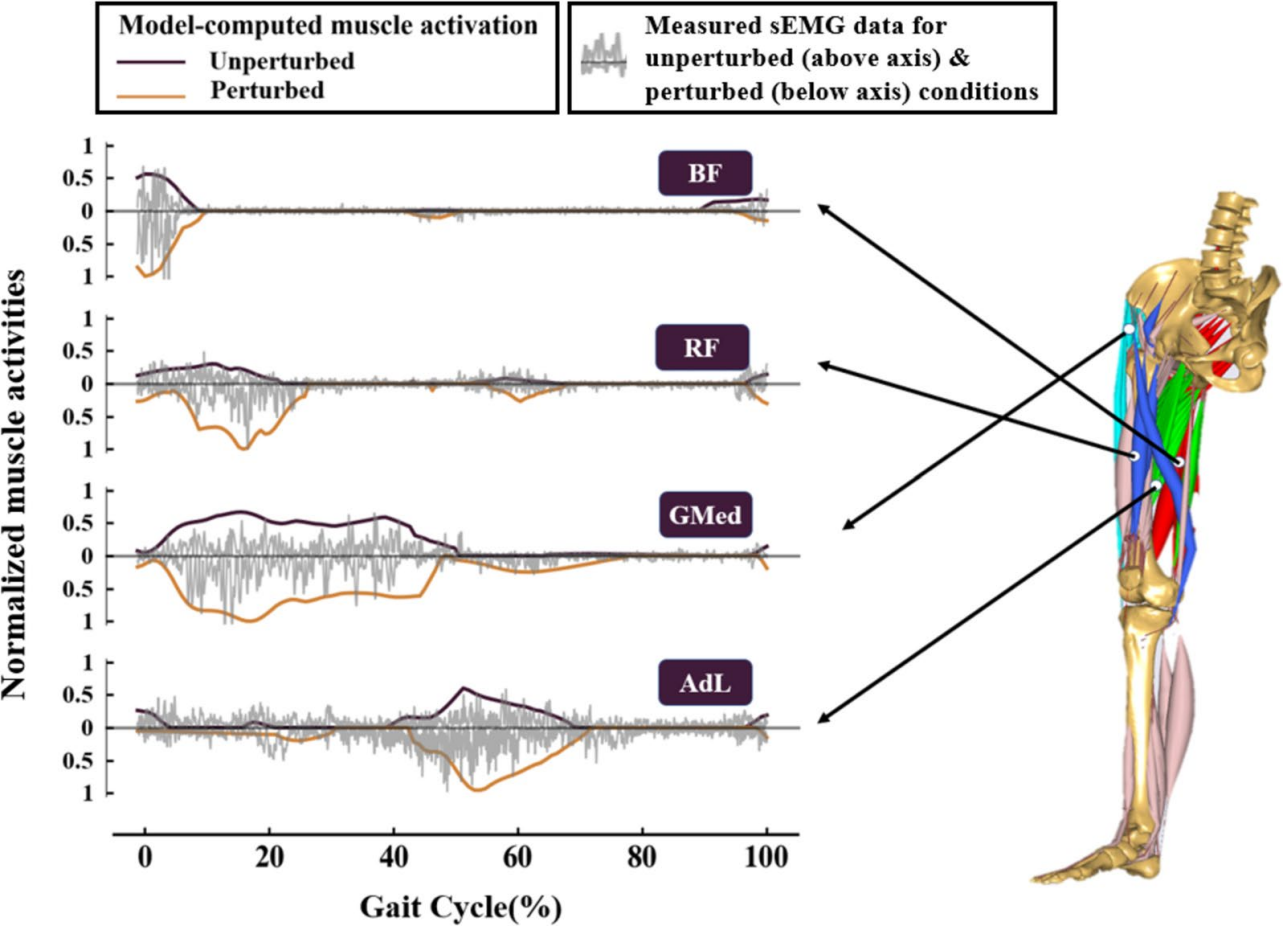
The comparison of changes in hip muscle activities between the model-computed results and the measured sEMG recordings during perturbed and unperturbed walking conditions in one random instance

As shown in Fig. 4, the simulated muscle activation in perturbed and unperturbed walking conditions was generally consistent with simultaneous recordings for four major chosen surface muscles. And Table 1 provided a summary of the correlation analysis results between model-simulated and experimental-measured muscle activity. All muscle activities measured by the sEMG device were significantly correlated with those computed by the subject-specific NMS (*p* < 0.05). The correlations between model-computed and experimental-measured results were strong for BF, RF and GMed, while the ADL only showed a moderate correlation.

**Table 1:** The average correlation coefficient between measured and simulated muscle activity for all experiment trials

Walking condition	BF	RF	GMed	ADL
Unperturbed	0.94	0.88	0.88	0.68
Perturbed	0.90	0.79	0.85	0.49

### Hip kinematics in unperturbed/perturbed walking

**Fig. 5:**
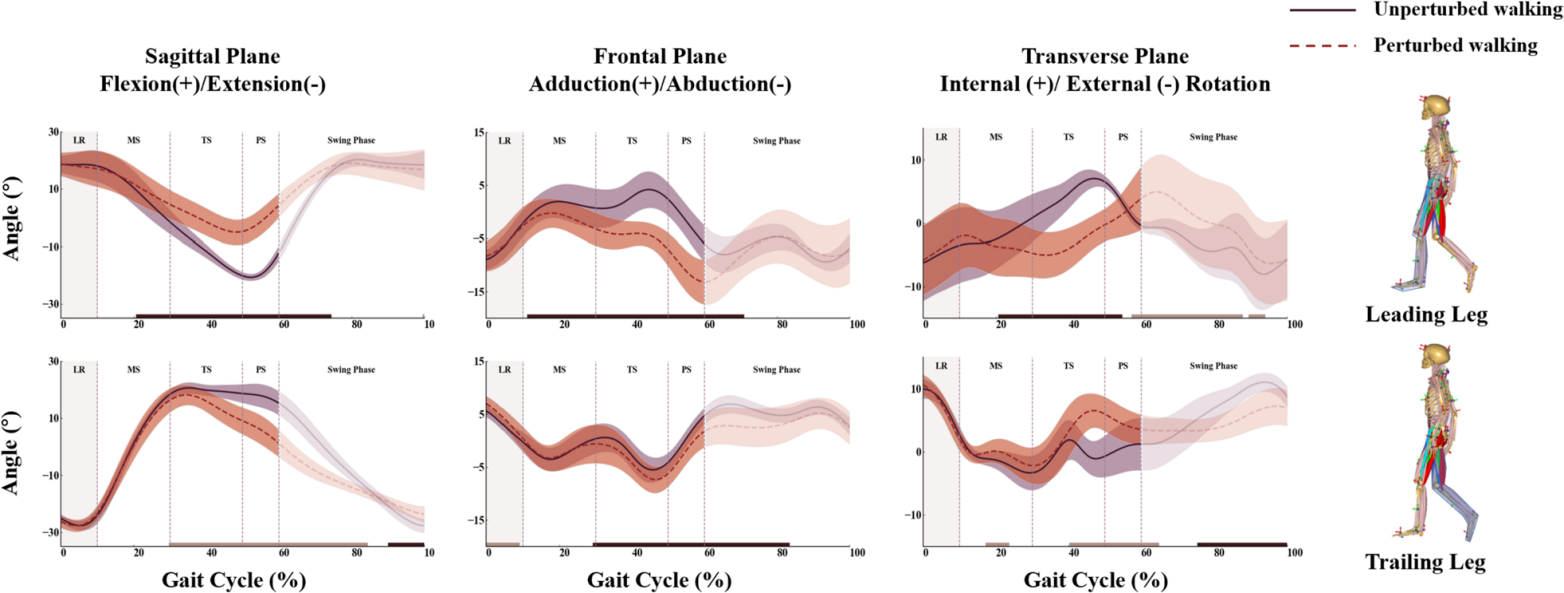
The kinematic patterns of the bilateral hip joints for all subjects during perturbed and unperturbed walking conditions. Gait phases were divided into loading response (LR), mid-stance (MS), terminal stance (TS) and pre-swing (PS) and swing phase based on the gait cycle of the leading leg. The left shaded area in the graph indicated the perturbation duration. Note in the bottom of the graph, solid black (significantly increased) and grey lines (significantly decreased) indicated the period where significant differences in joint angles (α ≤ 0.05) existed for the perturbed as compared against the unperturbed walking conditions

As shown in Fig. 5, compared to the unperturbed walking, the leading leg showed reduced sagittal motion starting from midstance and continuing into the swing phase (20.6–74.4% of the gait cycle, *p* < 0.05), increased abduction during single limb stance phase and pre-sewing phase (10.6–71.4%), as well as increased external rotation starting from midstance to the pre-swing phase (20.6–54.8%) due to lateral perturbation. Apart from that, the trailing leg reduced hip flexion (29.1–84.9%), and increased abduction (29.1–83.4%) starting from terminal stance to swing phase, and decreased internal rotation from terminal stance to pre-swing phase (40.2–65.3%).

### Hip kinetics in unperturbed/perturbed walking

**Fig. 6:**
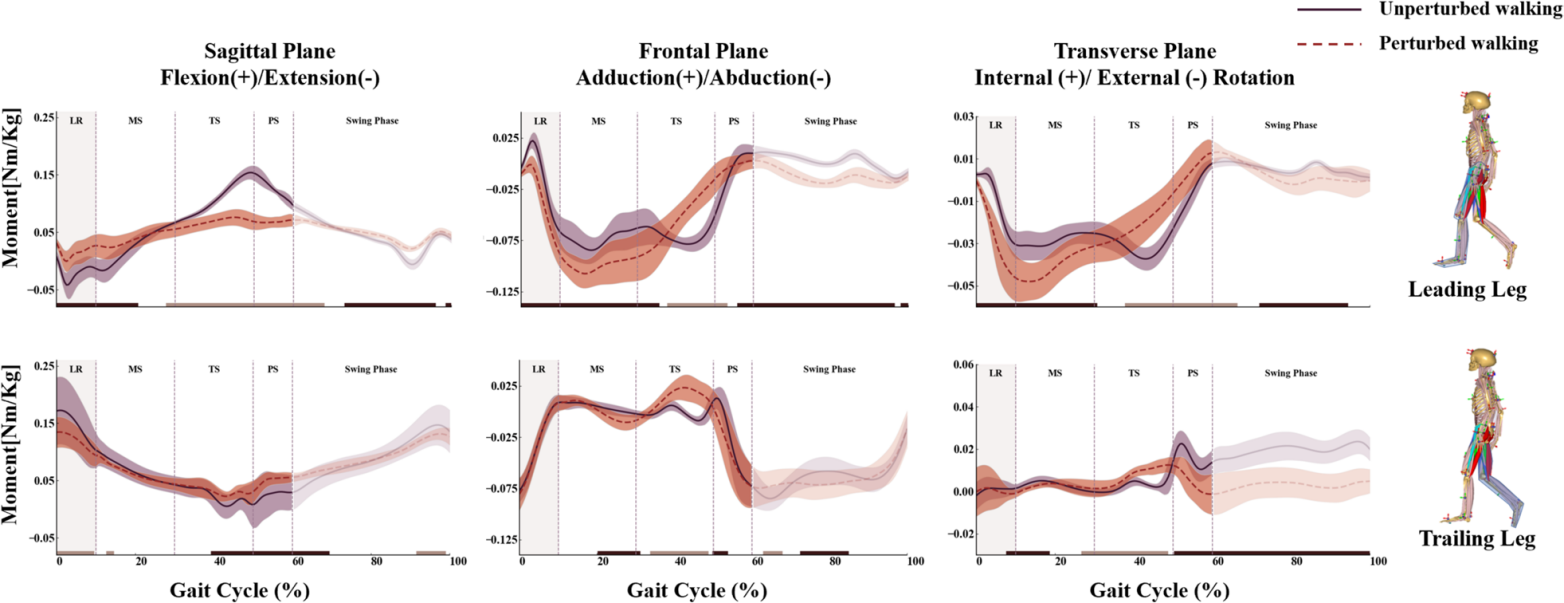
The kinetic patterns of the bilateral hip joints for all subjects during perturbed and unperturbed walking conditions. Note in the bottom of the graph, solid black (significantly increased) and grey lines (significantly decreased) indicated the period where significant differences in joint moments (α ≤ 0.05) existed for the perturbed as compared against the unperturbed walking conditions

Hip adaptive changes in kinetics were shown in Fig. 6. Compared to the unperturbed walking group, in terms of changes in hip kinetics in the sagittal plane, the leading leg had increased hip flexion moment (HFM) during loading response and midstance (0–20.6% of the gait cycle, *p* < 0.05) and decreased HFM during terminal stance and pre-swing phase (27.6–67.8%). For the frontal plane, the leading leg exhibited different hip adaptive changes during the single limb stance phase, with increasing hip adduction moment (HAM) during loading response and midstance (0–35.7%) and decreasing during terminal stance (37.7–55.3%). For the transverse plane, the leading leg increased external rotation moment (HEM) during loading response and midstance (0–30.7%) and decreased starting from terminal stance to pre-swing phase (37.7–66.3%).

The trailing leg showed decreased HFM during the loading response (0–9.0%), and increased HFM starting from terminal stance to swing phase (39.2–69.3%). For the frontal plane, the trailing leg also exhibited different hip adaptive changes during the single limb stance phase, increased HAM during midstance (20.1–31.7%) while decreased during terminal stance (33.7–48.7%). For the transverse plane, the trailing leg increased HEM during midstance (8.0–18.1%) and decreased during terminal stance (30.1–48.7%) as well.

### Muscles Coactivation Index (CI) for bilateral hip joints

**Fig.7:**
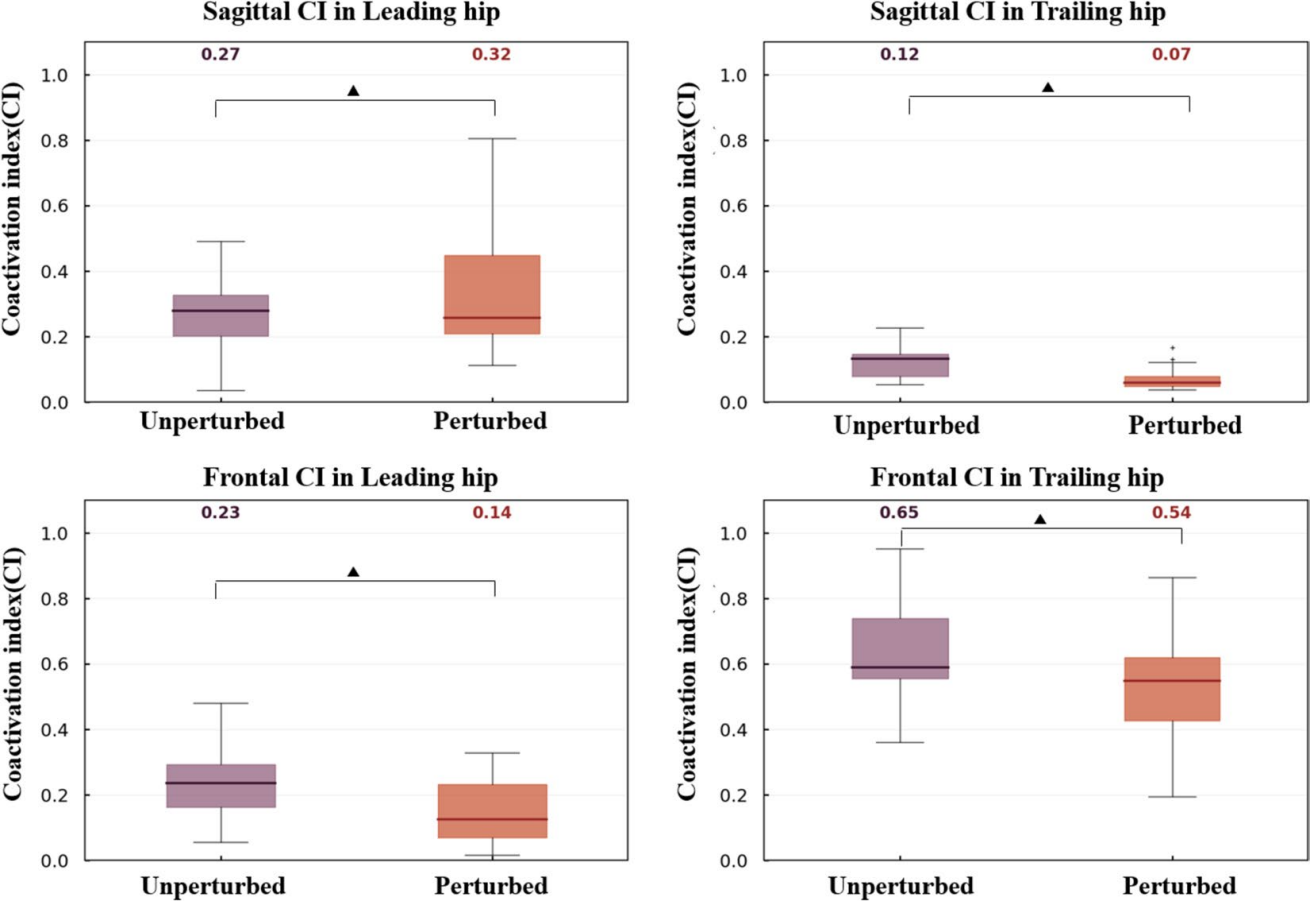
Effects of perturbation forces on the muscle coactivation index (CI) of the bilateral hip joints in the frontal and sagittal planes during step time. “” indicated the significant difference between perturbed walking and unperturbed walking

Agonist and antagonistic muscle groups' coactivation index of the hip joint changed significantly during step time due to lateral perturbation (Fig. 7). Compared to the unperturbed walking, the trailing leg reduced abductor and adductor muscles CI and flexor and extensor muscles CI (*p* < 0.05). And the leading leg had decreased abductor and adductor muscles CI (*p* < 0.05), while increased flexor and extensor muscles CI (*p* < 0.05).

## Discussion

This study aimed to develop subject-specific neuromusculoskeletal models (NMS) to investigate adaptive changes in bilateral hip mechanics due to unexpected perturbation. For the hip kinematics, bilateral hip joints had reduced sagittal-plane motion, while increased frontal and transversal-plane motion during step time. In terms of the kinetic responses, the bilateral hip showed distictive gait phase-dependent features. In particular, the bilateral hip joints had increased abductor moment during the middle stance (~ 10–25% gait cycle), but reduced joint moment in the frontal plane at the terminal stance (~ 40–55%). And muscle CI of the bilateral hip in the sagittal and frontal plane was decreased at the perturbed walking conditions, except for the leading leg in the sagittal plane.

The results suggested that healthy people tend to adopte a coordinated bilateral hip control strategy to maintain balance after lateral perturbation. The kinematics of the bilateral hip suggested that subjects had reduced sagittal movement by reducing flexion, meanwhile increasing frontal movement by increasing hip abduction and external rotation for unexpected lateral perturbation. The dynamic stability of gait depends on the intricate interplay between moving horizontal CoM trajectory and moving BoS, which is achieved through rapid and precise foot placements. The adaptive changes of hip mechanics seemed to be associated with the foot control strategy by increasing foot width and reducing step length [7], which aimed at expanding the width of BoS to conquer the unusual increase in lateral displacement of the CoM. It appears that the frontal plane adaptive changes in hip mechanics were consistent with a previous study conducted on a treadmil [33]. However, we observed differences in sagittal plane biomechanical responses. Specifically, our study revealed a reduced hip flexion moment in the leading leg and hip flexion in the trailing leg, in contrast to the previous treadmill-based study, which reported an increased hip flexion moment and no significant hip flexion difference. Such discrepancies could be attributed to the compelled propulsion and sensory conflicts induced by the fixed speed of the treadmill. [34]. Maintaining gait stability after lateral perturbation requires an adequate response in all three anatomical planes of motion. To the best of our knowledge, few studies have focused on the transverse plane motion of the hip. Our results revealed that the leading leg increased external rotation during the single limb support phase. And it was reported that the pelvis rotation assists in foot placement control [35], which indicated that the hip external rotation could also contribute to the foot adjustment due to lateral perturbation.

In addition to peak angle and moment differences, phase-dependent features revealed that the balance strategy of the bilateral hip joints was well-coordinated in different periods in gait. The midstance and terminal stance showed opposite hip kinetic responses for the leading leg’s flexor, abductor, external rotational moments and trailing leg’s abductor and external rotational moments. Based on the simplifying assumptions of the inverted pendulum model for balance control [2], the increased CoM velocity at the midstance and loading response phase led to reduced margin of stability (MoS) in the ML direction, which would increase the risk of falls due to the perturbation. And the MoS might decrease to zero or even negative values would not immediately lead to a fall, but would require corrective and fast actions to prevent falling [36]. Moreover, Wang’s study revealed that CoM’s position and velocity at midstance could explain an 80% variance in foot placement [37]. And its statistical findings revealed a pelvis mediolateral excursion of 1 cm corresponds to a 2 cm increase in base of support (BoS) width, while a velocity increase of 1 cm/s corresponds to a 0.44 cm BoS width increment. Thus, the increased hip joint moment at midstance could be associated with rapid foot placement control in respone to the excessive excursion of CoM. The bilateral hip demonstrated a decrease in abductor moment during terminal stance, preventing further expansion of step width. And The adoption of wider steps incurs an increase in metabolic energy cost due to the additional mechanical work required to redirect the motion of the CoM [38]. Thus, the biphasic modulation of bilateral hip abductor moment demonstrated the trade-off between gait stability and metabolic energy expenditure.

It is interesting to note that changes in hip joint moments during midstance and terminal stance could be associated with the modulation of muscle coactivation levels. The hip net joint moment was generated by optimizing the coactivation of the agonist and antagonist muscles. The increased muscles CI was characterized as a compensatory mechanism to enhance joint stability through increasing joint stiffness [18]. Thus, the increase of coactivation of hip flexors and extensors of the leading leg could contribute to enhanced joint stability during the weight acceptance phase and reduce the further forward movement of the trunk. But the excessive muscle CI increased postural rigidity at the same time inevitably which may inhibit smooth joint motion, restrict dynamic performance and increase energy cost [39]. Subjects who walked with higher ankle muscle co-contraction were predisposed to experience less severe slips when encountering an unexpectedly slippery floor [40]. The elderly often showed a higher muscle coactivation index than the younger [41] to compensate for the many neuromotor impairments associated with aging. And the elderly who experienced perturbation-based balance gait training showed reduced muscle CI and risk of falling significantly [42]. These findings proved our results to some extent and revealed that lower muscle CI in the frontal plane of the bilateral hip and sagittal plane of the trailing leg could be more beneficial for rapid foot placement control. In general, different muscle coactivation changes of the bilateral hip may be the result of a balance-related compensatory mechanism, which aimed at reducing trunk forward movements and increasing the MoS in ML effectively.

The subject-specific neuromusculoskeletal model was proven to be useful in determining activation patterns of the major hip muscles during perturbed walking. The computed muscle activity compared well with the measured EMG data for nearly all major hip muscles, and such agreement seemed to be consistent with previous studies [23, 27]. However, some discrepancies were noted for the hip adductor (ADL). According to the measured sEMG signals, the ADL was activated during the single limb stance phase (0–50% gait cycle), while the simulated ADL activation pattern suggested a slightly different phase activation pattern (0—5% and 18—30% of gait cycle), where some delays were visible. The difference between model-simulated and experimentally-measured EMG signals of ADL can be attributed to modelling simplifications. The muscle activation is under the exquisite control of the central nervous system, which could not be simply described by a single muscle recruitment algorithm. And, the ADL arises from the pubis inferior to the pubic crest and lateral to the pubic symphysis. Due to the narrow surface area exposed by the muscle bundle, cross-talk effects of the EMG signals from other muscles are also inevitable.

A subject-specific NMS model for hip adaptive mechanics is valuable for guiding balance assistive device design and active intervention strategies. Variations in bilateral hip kinematics in multi-anatomic planes emphasize the need for a multi-degree of freedom mechanisms [5]. The differences in the muscle CI further validate the feasibility of modulating joint stiffness to enhance gait stability [43]. The NMS model can also be integrated with the assistive device for virtual prototyping, optimizing control algorithm, and reducing design iterations [24]. Additionally, therapists can use the model to evaluate the efficacy of various balance training methods over time and make necessary adjustments for an intended treatment program [44].

There are limitations in this study. First, the feasibility of the subject-specific musculoskeletal model was only validated with a relatively small number of healthy subjects, which may inhabit statistical robustness. Additionally, owing to the design limitations of the customized gait-perturbing device, the perturbation maybe direction-predictable which could induce anticipatory postural response to some extent. Due to these limitations, future experiments should undertake more intricate perturbations (e.g., involving variations in timing) with a larger number of subjects to enhance the investigation of the hip adaptive response in balance recovery.

## Conclusion

To the best of our knowledge, it was the first attempt to use subject-specific neuromusculoskeletal models to determine the adaptive responses of bilateral hip joints during overground walking. The analysis of phase-dependent changes in bilateral hip abductor moment revealed a trade-off strategy between gait stability modulated by hip abduction and energy expenditure. Utilizing subject-specific neuromusculoskeletal models (NMS), alterations in hip muscle CI in multiple anatomical planes were determined. The bilateral hip joint muscle's CI in both sagittal and frontal planes were found to decrease during perturbed walking, except for the leading leg in the sagittal plane. These results suggest that bilateral hips may adopt decoupled balance recovery strategies in response to unexpected lateral perturbations, with an emphasis on enhancing joint stability in the sagittal plane while facilitating rapid foot placement control in the frontal plane during different gait phases. The study also proved the feasibility of using NMS as a non-invasive method for analyzing muscle functions during perturbing gait conditions.

### Supplementary Information

**Appendix I.** The normality test and paired t-test for muscle CI in different movement planes were summarized in the following table.

**Appendix II.** The hardware design and motor control software for the perturbation device.

## Data Availability

The datasets used and analyzed during the current study are available from the corresponding author on reasonable request.
