# Supplementary material for: A neuromusculoskeletal modelling approach to bilateral hip mechanics due to unexpected lateral perturbations during overground walking
Source: BMC Musculoskelet Disord. 2023 Oct 2;24:775. doi: 10.1186/s12891-023-06897-7 (PMC10544490; doi:10.1186/s12891-023-06897-7)
Supplement: Supplementary file 1 — Appendix I. The normality test and paired t-test for muscle CI in different movement planes were summarized in the following table. [file 12891_2023_6897_MOESM1_ESM.docx]

Table. 1 Normality test and paired t-test for muscle CI in different Movement planes were summarized in the following table.

| Variable | | Perturbed walking | | Unperturbed walking | | S-W test | Paired t-test |
| --- | --- | --- | --- | --- | --- | --- | --- |
|  |  | Mean | SD | Mean | SD |  |  |
| Leading hip | Sagittal | 0.27 | 0.09 | 0.32 | 0.17 | p<0.05 | p<0.05 |
|  | frontal | 0.23 | 0.08 | 0.14 | 0.08 | p=0.90 | p<0.05 |
| Trailing hip | Sagittal | 0.12 | 0.03 | 0.07 | 0.02 | p<0.55 | p<0.05 |
|  | frontal | 0.65 | 0.15 | 0.54 | 0.13 | p=0.55 | p<0.05 |
